# Development of MWCNTs/MXene/PVA Hydrogel Electrochemical Sensor for Multiplex Detection of Wound Infection Biomarkers

**DOI:** 10.3390/mi17020209

**Published:** 2026-02-03

**Authors:** Qihang Li, Jia Han, Ting Xue, Yuyu Bu

**Affiliations:** National Key Laboratory of Wide Bandgap Semiconductor Devices and Integrated Technology, School of Microelectronics, Xidian University, Xi’an 710071, China; 23111213597@stu.xidian.edu.cn (Q.L.);

**Keywords:** chronic wound infection, electrochemical sensor, composite hydrogel, multiplex detection

## Abstract

To address the clinical urgency of simultaneously monitoring multiple biomarkers in chronic wound infections, this study presents the innovative development of an electrochemical sensor based on a MWCNTs/MXene/PVA composite hydrogel. A dual-channel conductive network is constructed via the electrostatic self-assembly of the two-dimensional material MXene and multi-walled carbon nanotubes (MWCNTs). This strategy not only enhances the charge transfer efficiency but also effectively suppresses the aggregation of MWCNTs and exposes the electrocatalytic active sites. Additionally, the thermal annealing process is incorporated to facilitate the ordered arrangement of polyvinyl alcohol (PVA) nanocrystalline domains, strengthening the hydrogen bond-mediated interfacial adhesion and resolving the issues of hydrogel swelling and delamination. The detection limit (LOD) of the optimized sensor (MWCNTs_0.6_/MXene_0.4_/PVA) for pyocyanin (PCN) within complex biological matrices, including phosphate-buffered saline (PBS), Luria–Bertani (LB) broth, and saliva, was decreased to a range of 0.84~0.98 μM. Leveraging the disparities in the characteristic oxidation potentials (ΔE > 0.3 V) of PCN, uric acid (UA), and histamine (HA) in simulated wound exudate (SWE), the multi-component synchronous detection functionality of the non-specific sensor was expanded for the first time. This study offers a high-precision and multi-parameter integrated approach for point-of-care testing (POCT) of wound infections.

## 1. Introduction

Chronic wound infection poses a formidable challenge in clinical nursing. Among various pathogens, Pseudomonas aeruginosa (PA) has emerged as the primary causative agent of non-healing wounds. This is attributed to its remarkable drug resistance and proficient biofilm formation capabilities [1]. The dynamic biomarkers, namely PCN, UA, and HA in wound exudate, respectively, serve as characteristic indicators of the colonization of PA [2], metabolic anomalies [3,4], and the status of tissue repair [5,6]. Among these biomarkers, PCN is a signature secondary metabolite of PA. When the UA concentration deviates from the reference range of healthy wounds (200~750 μM), it can signify pathogen infection (<220 μM) or tissue necrosis (>750 μM). Abnormal HA concentration is closely associated with healing impairments (too low) or the risk of allergy/shock (too high). Consequently, the simultaneous detection of these biomarkers is of paramount importance for early infection warning and curative effect assessment. Traditional detection methods, such as high-performance liquid chromatography [7] (HPLC) or spectrophotometry [8], necessitate intricate sample pretreatment procedures and struggle to achieve simultaneous analysis of multiple indicators, thereby impeding the real-time evaluation of wound status.

Electrochemical sensors demonstrate substantial advantages in the realm of wound monitoring, attributable to their rapid response, portability, and high sensitivity [9]. Current study endeavors predominantly center on the detection of single markers. Hunter J et al. [10] devised a PCN sensor grounded on a screen-printed electrode, achieving an LOD of 1.9 μM. Pannawich et al. [11] fabricated a PCN molecularly imprinted sensor on a screen-printed carbon electrode (SPCE), with an LOD of 0.74 μM. These methodologies offer viable technical approaches for detection during the early phase of infection. Nevertheless, the relatively inferior biocompatibility of the materials restricts the direct utilization of these sensors in complex real biological specimens [12,13], such as wound exudate and saliva. In recent years, hydrogel materials have offered novel concepts for broadening the application of electrochemical sensors within biological environments, attributed to their outstanding biocompatibility, flexibility, and tissue adhesion capabilities [14,15,16,17]. Wei et al. [18] developed a “honeycomb-like” hydrogel electrochemical biosensor via 3D printing technology for the assessment of the combined toxicity of mycotoxins. Ernesto et al. [19] constructed an ultra-thin patch hydrogel sensor for the rapid detection of lactate in interstitial fluid (LOD = 0.15 mM). Wang et al. [20] devised a multifunctional Pd@Au nanoframework hydrogel with the aim of detecting UA through a chemo-photothermal approach for on-site monitoring of wound infections and facilitating wound healing. However, within a solution environment, the hydrogel modification layer is susceptible to a significant reduction in the interfacial adhesion force with the conductive substrate owing to swelling. Simultaneously, its intrinsic issues of inadequate electrical conductivity and poor stability severely impede the reliability of signal transmission and the long-term stability of the sensor [21,22]. The electrochemical sensor based on silane-functionalized MXene-PEGDA composite hydrogel developed by Yang et al. exhibits high sensitivity toward dopamine and uric acid (with LOD of 2.55 μM and 25.11 μM, respectively), but its detection range for UA does not cover clinically relevant concentrations [23]. Consequently, the development of a novel hydrogel sensor featuring high electron conductivity, robust interfacial adhesion stability, and the capacity to concurrently resolve multiple targets has emerged as a crucial breakthrough point for advancing the development of POCT technology for wound surfaces.

In light of the abovementioned issues, this study puts forward a three-dimensional composite hydrogel electrochemical sensor (MWCNTs/MXene/PVA) to concurrently tackle the aforesaid challenges via the strategies of material microstructure design and interfacial synergetic regulation. An “interlayer-out-of-layer” dual-path conductive network is constructed using MXene and MWCNTs. During the optimization of carrier transport channels, MXene not only inhibits the aggregation of MWCNTs but also exposes active sites, thereby increasing the electrochemical active area of the electrode to 1.32 times the reference value. Moreover, a thermal annealing process is employed to induce PVA to form ordered nanocrystalline domains. By strengthening the multiple hydrogen bond interactions between polymer chains and the rigid substrate, the interfacial adhesion is enhanced, resolving the problem of mechanical peeling under the swollen state. The research revealed that the optimized electrochemical sensor (MWCNTs_0.6_/MXene_0.4_/PVA) exhibits an impressively low detection limit for PCN, ranging from 0.84 to 0.98 μM in PBS, LB broth, and saliva. This detection limit is 73% lower than that of the single MWCNTs system. Innovatively, this sensor capitalizes on the disparities in the characteristic oxidation potentials of three biomarkers in SWE to successfully accomplish highly selective simultaneous detection of multiple components. It overcomes the cross-interference constraint imposed by the overlapping oxidation potentials (ΔE < 0.3 V) of traditional non-specific sensors. When combined with square wave voltammetry (SWV), it showcases stable sensing of the main target substance, with the interference of the varying concentration of coexisting substances being less than 6% (RSD). This study offers a high-precision, multi-parameter integrated solution for the dynamic monitoring of wound infections and facilitates the clinical translation of flexible POCT devices.

## 2. Experimental Section

### 2.1. Preparation of the MWCNTs/MXene/PVA Electrochemical Sensor

The details of materials used in this work are provided in the Supplementary Materials.

Initially, aqueous solutions of Hexadecyl trimethyl ammonium Bromide (CTAB) at varying concentrations (ranging from 0.2 to 1.0 wt%) were meticulously prepared to serve as the dispersion medium. Subsequently, carboxylated multi-walled carbon nanotubes (COOH-MWCNTs) and Ti_2_CT_x_ MXene nanosheets with diverse mass ratios (MWCNTs:MXene = 0.9:0.1, 0.8:0.2, 0.7:0.3, 0.6:0.4, 0.5:0.5, with a combined total mass of 0.10 g) were precisely added to 10 mL of the CTAB solution. The resulting mixture was mechanically agitated at a speed of 1000 r/min for 30 min, followed by 1 h of ultrasonic treatment in an ice bath (power: 300 W, pulse on/off cycle: 5 s/5 s) to achieve a homogeneous and stable MWCNTs/MXene composite dispersion. Subsequently, 0.3 g of PVA powder was introduced into this dispersion (resulting in a final concentration of approximately 3 wt%). The mixture was then continuously stirred at 600 r/min in a 95 °C water bath for 1 h to guarantee the complete dissolution of PVA. Finally, a series of MWCNTs/MXene/PVA composite hydrogel precursors were fabricated and stored at room temperature under sealed and light-protected conditions. For the sensor assembly, pre-treated indium tin oxide (ITO) glass serves as the working electrode substrate. The ITO substrate is sequentially ultrasonically cleaned in a glass cleaning agent, anhydrous ethanol, and ultrapure water at 42 °C for 15 min each. After that, it is dried using a high-purity nitrogen gas stream. Then, 5 μL of the MWCNTs/MXene/PVA composite hydrogel precursor solution is pipetted and evenly dropped onto the clean ITO surface (effective area: 0.25 cm^2^), followed by drying at room temperature. Subsequently, the modified electrode undergoes 3 cycles of freeze-thaw treatment (each cycle consists of freezing at -20 °C for 30 min and thawing at 25 °C for 30 min). The optimal freeze-thaw cycle can establish a hydrogel network with suitable crystallinity to ensure strong adhesion and minimal swelling. Finally, the electrode is placed in a forced-air drying oven for 1 h to complete the fabrication of the MWCNTs/MXene/PVA composite hydrogel sensor (Figure S1). An electrochemical workstation and a three-electrode system are employed to conduct the subsequent electrochemical tests of the sensor.

### 2.2. Instruments

The microscopic morphology and elemental mapping of the sensor were examined using a scanning electron microscope (SEM, GeminiSEM 360, Zeiss, Jena, Germany) and an optical microscope (OM, Lab A1, Zeiss, Jena, Germany). The surface micrographics of the electrode were characterized by an atomic force microscope (AFM, Dimension Icon, Bruker, Billerica, MA, USA) at a scan frequency of 1.58 Hz. The chemical composition and crystal structure of the electrode were analyzed via X-ray diffraction analysis (XRD, D8 Advance, Bruker, Billerica, MA, USA), X-ray photoelectron spectroscopy (XPS, ESCALAB Xi+, Thermo Scientific, Hillsboro, OR, USA), and Fourier Transform infrared spectroscopy (FT-IR, IRTracer 100, Tsushima Co., Kyoto, Japan). Wide angle X-ray scattering (WAXS, Xeuss 2.0, Xenocs, Grenoble, France) was employed to characterize the degree of integration of the two phases.

Electrochemical performance measurements were performed at a ChenHua Electrochemical Workstation (CHI660E, Chenhua Instrument Co., Ltd., Shanghai, China) in a traditional three-electrode cell containing 1x phosphate-buffered solution (PBS, pH 7.3) with Ag/AgCl as reference electrode and a piece of platinum as counter electrode, respectively. All the potentials described in this paper are transformed into values referenced to the reversible hydrogen electrode. The conversion formula is as follows:

E_(RHE)_ = E_(RE)_ + E_(Ag/AgCl)_ + 0.0592 × pH (1)

Among these, E_(RE)_ represents the reference electrode potential. For the Ag/AgCl electrode filled with saturated potassium chloride solution, the value of E_(RE)_ is 0.1971 V. E_(Ag/AgCl)_ denotes the experimental measurement potential when the Ag/AgCl electrode is used as the reference electrode.

Electrochemical impedance spectroscopy (EIS) was completed in a solution of 0.1 M KCl containing 5 mM [Fe(CN)_6_]^3−/4−^ at a Zahner Zennium pro Electrochemical Workstation (Zahner, Germany) in the same three-electrode cell. The detection limit (LOD) of sensor can be calculated by Equation (2):

(2) $$LOD=\frac{3S_{b}}{K}$$

where *S_b_* is standard deviation of blank sample, *K* is slope of the calibration curve.

## 3. Result and Discussion

### 3.1. Optimization of Preparation Parameters for the MWCNTs/MXene/PVA Electrochemical Sensor

MWCNTs exhibit a strong tendency to aggregate readily. This is primarily attributed to the π-π stacking within the sp^2^ hybrid structure and the presence of van der Waals forces [24]. Consequently, the cationic surfactant CTAB is employed to facilitate stable dispersion. During the dispersion process, the positively charged head groups of CTAB are electrostatically anchored at the -COOH sites of MWCNTs. Simultaneously, the hydrophobic alkyl chains interact via van der Waals forces, jointly establishing a synergistic and stable structure characterized by electrostatic repulsion and steric hindrance. The MXene material (Ti_2_CT_x_) was synthesized by selectively etching the Al layers from the Ti_2_AlC MAX phase using HF at room temperature over a period of 24 h. This etching process introduces various terminal functional groups (–OH, –O, –F) on the MXene surface, imparting it with a negative charge. This enables electrostatic self-assembly with positively charged MWCNTs, thereby forming a stable composite structure. These functional groups can electrostatically self-assemble with positively charged MWCNTs to construct a composite system. As shown in Figure S2B,C, when the mass fraction of CTAB was 0.4%, the UV absorption reached its maximum at λ = 262 nm and exhibited the slowest normalized absorbance decay over 48 h, indicating the optimal dispersion performance [25]. When the amount of CTAB is insufficient, incomplete coverage results in the aggregation of MWCNTs. Conversely, when the amount is excessive, micellization induces the depletion effect, leading to the re-aggregation of MWCNTs. When the mass fraction of CTAB is 1%, the large-sized micelles exert a steric hindrance effect, which partially restores the dispersibility of MWCNTs. These results are consistent with the double-platform variation trend of CTAB in dispersing MWCNTs.

**Figure 1:**
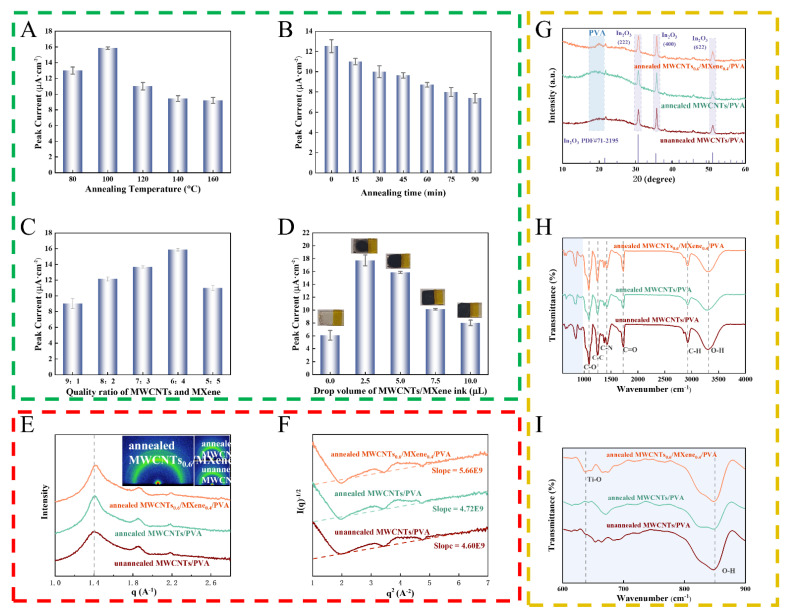
The present responses of the MWCNTs/MXene/PVA series sensors to 50 μM PCN under diverse conditions. (**A**) Varied annealing temperatures; (**B**) diverse annealing durations; (**C**) different doping levels of MXene; (**D**) distinct amounts of material modification on the ITO conductive substrate; Sensors of unannealed MWCNTs/PVA, annealed MWNCTs/PVA, and annealed MWCNTs_0.6_/MXene_0.4_/PVA; (**E**) GI-WAXS spectrum (the inset is the X-ray diffraction pattern); (**F**) Debye–Bueche curve and fitting results based on the GI-WAXS spectrum; (**G**) XRD patterns; (**H**,**I**) FT-IR patterns.

As depicted in Figure 1A,B, following freeze-thaw cycling, the PVA attains optimized crystallinity and an enhanced interface when annealed at 100 °C for 15 min. This phenomenon can be attributed to the fact that during low-temperature annealing (<100 °C), the hydrogel film experiences insufficient crystallization. This is due to the restricted mobility of molecular segments, which in turn gives rise to swelling instability. Conversely, during high-temperature annealing (>100 °C), excessive crystallization within the film leads to internal stress concentration, ultimately resulting in brittle fracture of the material. The investigation into the annealing time reveals that although the untreated sample exhibits a high initial response, significant swelling interference occurs during detection. Extending the annealing time can enhance the interface adhesion. However, an overly high crystallinity will impede the mass transfer of the analyte, thereby causing attenuation of the electrochemical response [26].

In Figure 1E, GI-WAXS tests provided conclusive evidence that annealing led to the preferential orientation of PVA nanocrystalline domains along the direction parallel to the substrate [27]. This phenomenon significantly enhanced the interfacial adhesion strength mediated by hydrogen bonds. In Figure 1G–I, XRD and FT-IR analyses demonstrated that following annealing, the crystallization peaks of PVA became more distinct. The intensities of the characteristic peaks at 3313 cm^−1^ (O-H stretching vibration) and 855 cm^−1^ (O-H bending vibration) decreased, suggesting a reduction in the free water content and an improvement in the crystallization perfection. Simultaneously, the emergence of the Ti-O characteristic peak at 638 cm^−1^ verified the successful incorporation of MXene [28].

Further optimization of the MWCNTs/MXene ratio was conducted. As depicted in Figure 1C, the 6:4 ratio demonstrates the optimal sensing performance. This phenomenon can be attributed to the fact that when the proportion of MXene is inadequate (<40%), the intercalated structure defects give rise to an incomplete conductive network. When the two materials are compounded in equal proportions, the MWCNTs fail to effectively function as an interlayer spacer, thereby impeding carrier transport. The investigation into the drop-casting volume reveals that 5 μL can achieve uniform coverage on the ITO substrate (coverage is incomplete when it is 2.5 μL). Excessive drop-casting leads to carrier migration delay and current saturation owing to the increased thickness of the hydrogel (Figure 1D). As evident from Figure 1F, for the finally fabricated MWCNTs_0.6_/MXene_0.4_/PVA composite system after the combined freeze-thaw and annealing treatment, the GI-WAXS scattering peak shifts to a higher q value, suggesting a reduction in the phase separation size between the materials. The optimized fitting slope validates the enhanced compatibility of the components, providing an ideal interfacial structure and mass transfer channels for high-performance sensors.

### 3.2. The Sensing Performance of the MWCNTs/MXene/PVA Electrochemical Sensor for PCN

In this study, the MWCNTs_0.6_/MXene_0.4_/PVA sensor featuring optimal sensing performance was employed to investigate the sensing performance of the target analyte PCN. Cyclic voltammetry (CV) tests (Figure S3) reveal that both the anodic and cathodic peak currents exhibit a linear relationship with the scan rate. The redox process of PCN on the MWCNTs_0.6_/MXene_0.4_/PVA sensor adheres to an adsorption-controlled mechanism, as evidenced by Equation (3):

(3) $$I_{p}=\frac{n^{2}F^{2}}{4RT}\upsilon A\Gamma *$$

Here, *υ* represents the CV scanning rate, *A* denotes the electrode area, and Γ* signifies the surface coverage of the adsorbed substance [29]. Consequently, all subsequent concentration gradient tests were conducted after the sensor had been incubated in PCN solutions of varying concentrations for an identical duration.

**Figure 2:**
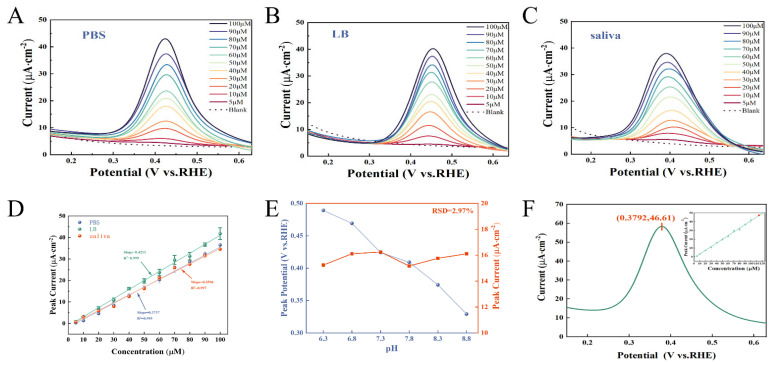
The SWV current responses of MWCNTs_0.6_/MXene_0.4_/PVA in different electrolytes. (**A**) PBS solution; (**B**) LB broth; (**C**) artificial saliva and (**D**) the standard curve; MWCNTs_0.6_/MXene_0.4_/PVA; (**E**) the current responses in PBS environments with different pH values (containing 50 μM PCN) and the oxidation peak potential of PCN; (**F**) the SWV current responses of the supernatant of PA medium (including PCN).

The SWV was employed to assess the sensing performance of the MWCNTs_0.6_/MXene_0.4_/PVA sensor for the target analyte PCN in diverse electrolyte environments. As depicted in Figure 2A, within PBS, PCN manifested a distinct oxidation peak at 0.424 V. Notably, its peak current increased substantially with the elevation of PCN concentration, spanning from 5 to 100 μM. The current–concentration fitting outcomes (Figure 2D) demonstrated an excellent linear relationship (R^2^ = 0.995), with an LOD of 0.94 μM, encompassing the clinical concentration range of PCN associated with PA infection. Similar oxidation behaviors of PCN were witnessed in LB broth (which simulated the growth environment of PA, as shown in Figure 2B) and artificial saliva (Figure 2C). The peak potentials were 0.454 V and 0.389 V, respectively, and the LOD were 0.84 μM and 0.98 μM respectively. In comparison with recent research findings, the sensor showcases superior sensitivity and lower LOD in three electrolytes. This performance demonstrates that MXene doping effectively enhances the sensor’s detection capabilities for PCN. Notably, the oxidation peak potential of PCN varies across different electrolytes (Figure 2A–C), suggesting that its oxidation behavior is influenced by pH. The experiment was conducted in PBS that simulated the pH environment (ranging from 7 to 9) of chronic wounds [30]. In this experiment, the concentration of PCN was fixed at 50 μM (Figure 2E). As the pH value increased, the oxidation peak potential shifted negatively. This phenomenon could be ascribed to the deprotonation of the acidic -OH groups within the PCN molecule, which in turn enhanced the electron delocalization. Notwithstanding the shift in potential, the RSD of the oxidation peak current was merely 2.97%. This result demonstrated that the sensor exhibited excellent robustness against pH variations in the testing environment. Figure 2F depicts the test results of the sensor in the supernatant of LB broth (pH = 7.9) subsequent to culturing PA. Based on the calibration curve presented in Figure 2D, the concentration of PCN was determined to be 113.23 μM. This value is in high agreement with the result obtained via the standard purification and weighing method (113 μM). These findings validate the accuracy and application potential of the sensor for detecting PCN in complex biological samples.

To comprehensively and systematically assess the impact of MXene doping on the sensing performance, this study carried out in-depth investigations on the anti-interference ability, stability, and reproducibility of the MWCNTs_0.6_/MXene_0.4_/PVA sensor. Specifically, glucose, ascorbic acid, urea, tryptophan, Na^+^, and K^+^ were added at the same molar concentration to a solution containing 50 μM PCN. As depicted in Figure S4A, the deviation of the current response was found to be within ±6%. Figure S4B reveals that after 19 days, the current response of the sensor still maintained 86.9% of its initial value. Moreover, as presented in Figure S4C, the RSD of the current response in independent batch experiments was less than ±5%. Collectively, these findings clearly demonstrate that the MXene composite system substantially enhances the overall performance of the sensor.

### 3.3. The Carrier Transport Mechanism of the MWCNTs/MXene/PVA Electrochemical Sensor

After verifying the outstanding PCN detection performance of the MWCNTs_0.6_/MXene_0.4_/PVA electrochemical sensor, it is of great significance to conduct an in-depth investigation into its internal carrier transport kinetic mechanism. This is essential for elucidating the underlying reason for the enhancement of the sensing performance brought about by MXene doping.

**Figure 3:**
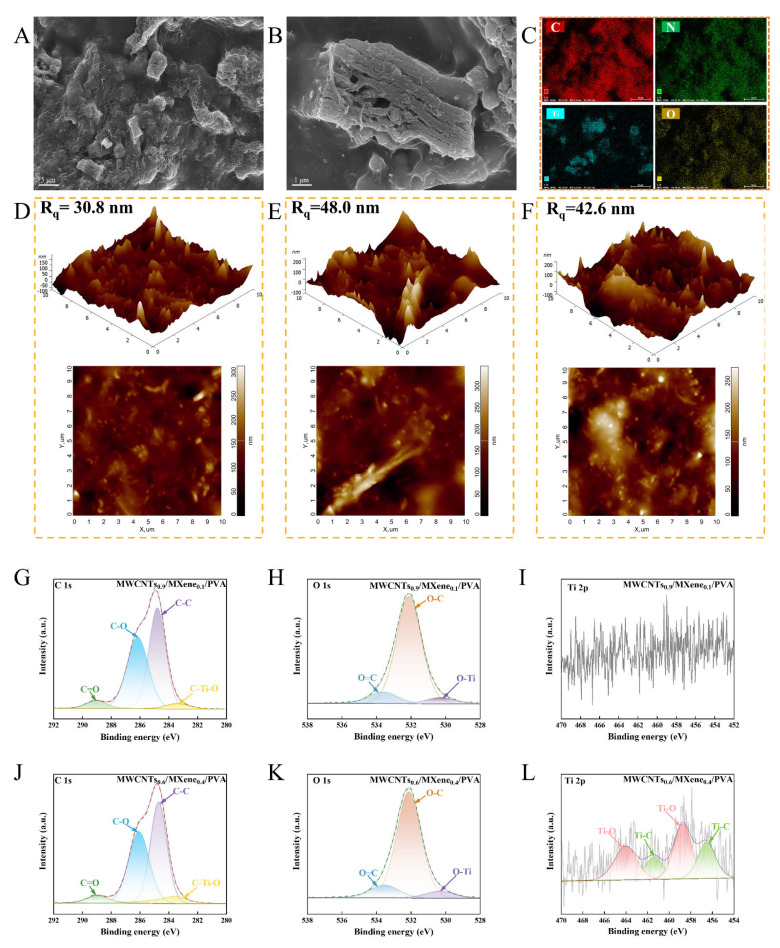
The sensor prepared from the MWCNTs_0.6_/MXene_0.4_/PVA lower layer solution. (**A**) SEM image with a scale of 5 μm; (**B**) SEM image with a scale of 1 μm; and (**C**) EDS surface scanning image; 3D and 2D AFM images of MWCNTs/MXene/PVA series sensors. (**D**) MWCNTs_0.9_/MXene_0.1_/PVA (**E**) MWCNTs_0.6_/MXene_0.4_/PVA; (**F**) MWCNTs_0.5_/MXene_0.5_/PVA; (**G**–**I**) C 1s, O 1s and Ti 2p spectra of MWCNTs_0.9_/MXene_0.1_/PVA; (**J**–**L**) C 1s, O 1s and Ti 2p spectra of MWCNTs_0.6_/MXene_0.4_/PVA.

During the preparation of the composite hydrogel system, a delamination phenomenon was witnessed, as can be seen in Figure 3A–C. In the lower layer solution, a distinct composite structure emerged between the multi-layered Ti_2_CT_x_ MXene and the tubular MWCNTs. EDS verified that the Ti element was concentrated in blocks on the surface of the sensor. Acting as a spacer, MWCNTs effectively suppressed the interlayer stacking of MXene [31,32,33]. Figure S5 demonstrates that the upper-layer solution leads to the fragmentation of MXene as a consequence of ultrasonic exfoliation, and the MXene is enwrapped by PVA. Only MWCNTs are exposed on the surface, and the Ti signal is undetectable. Although the conductivity of the lower-layer sample is so high that it may interfere with trace detection, the microscopic structure analysis discloses the anchoring mechanism of MWCNTs-MXene during electrostatic self-assembly.

The content of MXene exerts a significant influence on the surface morphology and roughness. As demonstrated by AFM characterization (Figure 3D,E), the MWCNTs_0.6_/MXene_0.4_/PVA system exhibits the highest roughness (Rq = 48 nm), featuring a continuous large-peak structure. This can be attributed to the three-dimensional anchoring of MWCNTs between and on the MXene layers. When the MXene content is relatively low, MWCNTs are discretely distributed (Rq = 30.8 nm). In contrast, in the equal-proportion system, a smooth peak structure is formed as a result of excessive embedding (Rq = 42.6 nm). Figure S6 demonstrates that the surface roughness of the sensor exhibits a positive correlation with the number of exposed multi-walled carbon nanotubes (MWCNTs). Specifically, the ratio of 0.6/0.4 is greater than 0.5/0.5, which is in turn greater than 0.9/0.1, thereby providing more active sites for the reaction. XPS analysis (Figure 3I,L) reveals that only the MWCNTs_0.6_/MXene_0.4_/PVA sample shows the characteristic Ti 2p peaks (Ti-O/Ti-C) [34]. Moreover, the content of C-Ti-O bonds in its C 1s spectrum is significantly higher than that of MWCNTs_0.9_/MXene_0.1_/PVA (Table S1). This finding confirms that MXene has successfully participated in the construction of the surface composite structure. When combined with the AFM morphology and EDS element distribution results, this evidence verifies the formation of a strong synergistic interface between MWCNTs and MXene.

**Figure 4:**
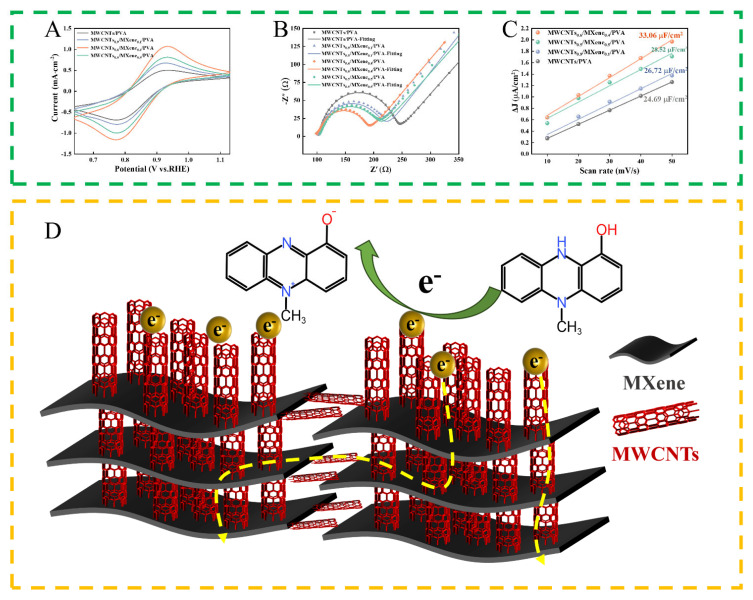
Electrochemical performance characterization of the MWCNTs/MXene/PVA series sensors was conducted in a 0.1 M KCl solution containing 5 mM [Fe(CN)_6_]^3-/4-^. (**A**) CV characterization; (**B**) EIS characterization and fitting outcomes; (**C**) graph of the current density difference in a series of sensors as a function of scan rate; (**D**) schematic diagram of the carrier transport mechanism of the MWCNTs_0.6_/MXene_0.4_/PVA sensor.

As depicted in Figure 4A, within the [Fe(CN)_6_]^3−^/^4−^ system, all MWCNTs/MXene/PVA sensors demonstrate reversible redox peaks. Among these sensors, the MWCNTs_0.6_/MXene_0.4_/PVA sensor exhibits an oxidation current of up to 1.04 mA, which implies its optimal electrical conductivity. EIS analysis (Figure 4B and Figure S8) further validates that it exhibits the lowest interfacial charge transfer resistance R_2_ and internal carrier transport resistance (R_1_ = 87.79 Ω). This can be ascribed to the fact that MXene facilitates charge separation and the MWCNTs/MXene composite structure optimizes the carrier channels [35]. As depicted in Figure 4C, upon the addition of MXene, the slopes of the MWCNTs/MXene/PVA series sensors all increase. Among them, the MWCNTs_0.6_/MXene_0.4_/PVA sensor demonstrates the largest slope (33.06 μF/cm^2^), suggesting that it possesses the greatest number of electroactive reaction sites on its surface. This can significantly enhance the adsorption of the target substance and the reaction kinetics [36].

By integrating the comprehensive characterization and testing results, this study uncovers the mechanism through which MXene enhances the performance of sensors. MXene and MWCNTs construct a three-dimensional heterostructure via electrostatic self-assembly. The high specific surface area of this heterostructure offers an ideal loading platform for MWCNTs. In turn, MWCNTs optimize the conductive network through a dual-space anchoring mechanism. As physical spacers, MWCNTs are intercalated between MXene layers, which effectively inhibits the π-π stacking of MXene sheets. This not only maintains the interlayer carrier transport channels but also exposes the shielded electrochemical active sites. Simultaneously, MWCNTs are anchored onto the surface and edges of MXene, thereby constructing an out-of-layer electron transport bridge across the sheets. Furthermore, electrochemical findings demonstrate that the mass ratio of MWCNTs to MXene plays a crucial and decisive role in the construction of the three-dimensional conductive network. A relatively low proportion of MXene can cause the fragmentation of the heterostructure and impede the carrier transport pathway. When they are compounded in equal ratios, an excessive number of MWCNTs are inserted into the interlayers, forming a dense intercalation structure. Nevertheless, the insufficient surface anchoring amount leads to the disconnection of the channels between the interlayers and the outer layers. The optimization mechanism of MWCNTs_0.6_/MXene_0.4_/PVA resides in its structural model (Figure 4D). The MWCNTs intercalated between the layers effectively expand the interlayer spacing, while the excess MWCNTs are firmly anchored on the outer surface of MXene. This facilitates the formation of a continuous and interconnected three-dimensional electron transport network within the longitudinal interlayer channels and the transverse surface channels. As a result, a low-impedance electron superhighway is established, thereby significantly enhancing the sensing response sensitivity of the system.

### 3.4. MWCNTs/MXene/PVA Electrochemical Sensor for Multiplex Detection of Wound Exudate

To assess the practical application potential of the developed MWCNTs_0.6_/MXene_0.4_/PVA electrochemical sensor within complex biological environments, the response characteristics of the sensor to UA and HA were initially quantified in a PBS environment (Figures S9 and S10). The scan rate-dependent experiment verified that the oxidation of UA is an irreversible process (only an anodic peak is detected), whereas HA demonstrates a reversible redox behavior (both anodic and cathodic peaks are evident). This finding aligns with the electrochemical characteristics of their molecular structures (purine ring vs. imidazole ring) [37,38]. The sensor exhibits excellent linear responses to UA in the concentration range of 200~900 μM and to HA in the range of 10~60 μM, with LODs of 66.67 μM and 3.34 μM, respectively. Significantly, this linear range precisely encompasses the clinically relevant concentrations.

The aforementioned results comprehensively illustrate that the developed MWCNTs_0.6_/MXene_0.4_/PVA electrochemical sensor showcases outstanding single-sensing capabilities for three biomolecules, specifically PCN, UA, and HA. In this research, the combined detection performance of the sensor was systematically explored. All the experiments were conducted within the SWE matrix to maximize the simulation of the real physiological environment.

**Figure 5:**
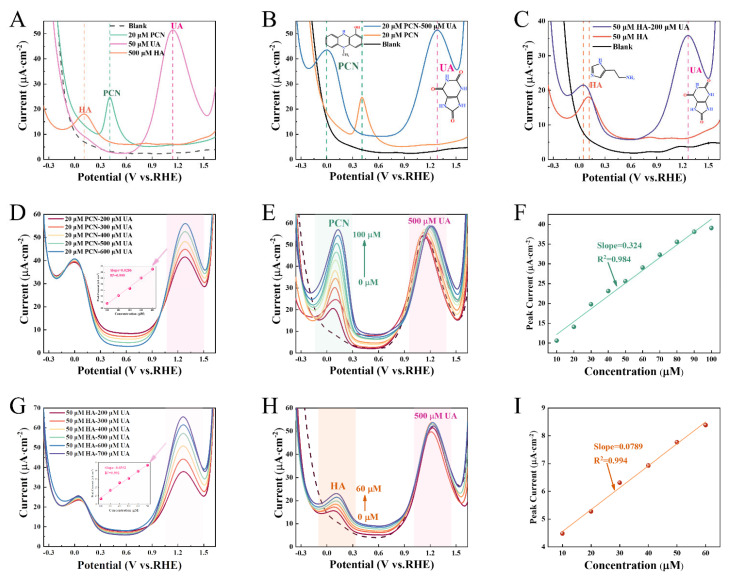
(**A**) Validation of the combined detection capacity of MWCNTs_0.6_/MXene_0.4_/PVA; (**B**,**C**) SWV current responses of MWCNTs_0.6_/MXene_0.4_/PVA under diverse conditions; (**D**) SWV current responses as the concentration of UA varies at a fixed concentration of PCN; (**E**,**F**) SWV current responses and their corresponding standard curves with a gradient of PCN concentration at a fixed UA concentration; (**G**) SWV current responses with the variation in UA concentration at a fixed concentration of HA; (**H**,**I**) SWV current responses and their standard curves with a gradient of HA concentration at a fixed UA concentration.

In Figure 5A, notable differences (ΔE > 0.3 V) were discerned in the characteristic oxidation potentials of PCN, UA, and HA in SWE. Specifically, the oxidation potential of PCN was approximately 0.404 V, that of HA was around 0.104 V, and UA exhibited a potential of approximately 1.139 V versus the reversible hydrogen electrode (RHE). Nevertheless, the disparity in the oxidation peak potential between PCN and HA was merely 0.3 V. Moreover, UA is solely soluble in an alkaline environment, and the oxidation potential of PCN diminishes in such an environment. Collectively, these characteristics augment the likelihood of peak overlap during the simultaneous detection of these three components. Consequently, in this study, binary systems of PCN-UA and HA-UA were established to assess the simultaneous detection performance of the sensor.

In the PCN-UA system (Figure 5B), the sensor demonstrates two distinct oxidation peaks at 0.404 V (PCN) and 1.28 V (UA). Analogously, in the HA-UA system (Figure 5C), the peaks at 0.114 V (HA) and 1.239 V (UA) are clearly discernible. Notwithstanding the shift in the peak potential of the main target substance, the change rate of its peak current was less than 6% (RSD), thereby corroborating an excellent synchronous detection capacity. Subsequently, to validate the anti-interference performance, the concentration of PCN was held constant while that of UA was varied (Figure 5D). Notably, there was no discernible alteration in the peak current fluctuation of PCN. When the concentration of UA was adjusted while keeping HA fixed (Figure 5G), or when the concentration of HA/PCN was changed while UA was maintained at a constant level (Figure S11B or S12B), the response current of the target substance remained stable as well. This evidence suggests that the detection of the main analyte by the sensor is not substantially influenced by the concentration variations in other coexisting substances, underscoring its reliable quantitative capabilities for specific markers within complex matrices. Furthermore, even at a high concentration of interfering substances (500 μM UA), the sensor retained excellent linearity for PCN (10~100 μM) and HA (10~60 μM) (Figure 5E,F,H,I). The LOD were 1.04 μM and 4.27 μM for PCN and HA, respectively. Notably, when PCN coexisted with UA, the slope of the standard curve decreased by approximately 19% relative to the single system, and the LOD for PCN increased to 81.76 μM (Figure S12D). According to the literature [4], it is hypothesized that this attenuation might be attributed to the metabolic consumption of UA by PCN. This implies that in practical applications, the impact of biomolecular interactions on the effective concentration of the target substance should be taken into account.

This finding for the first time reveals that a single non-specific sensor (MWCNTs_0.6_/MXene_0.4_/PVA) is capable of achieving the simultaneous detection of crucial infection markers in wound exudate, including PCN, UA, and HA, without the aid of antibodies or enzymes. Furthermore, the entire process necessitates only 4 min of adsorption and enrichment time. This single-sensor multi-analyte detection strategy substantially enhances efficiency and offers an innovative and practical technical solution for the development of POCT devices aimed at real-time monitoring of wound infections and healing assessment.

## 4. Conclusions

In this research, a thermally annealed enhanced MWCNTs/MXene/PVA composite hydrogel-based electrochemical sensor was successfully fabricated to address the clinical requirement for the simultaneous monitoring of multiple markers in chronic wound infections. Leveraging the synergistic effect of MXene/MWCNT’s dual-channel conductive network and the thermal annealing-induced ordering of PVA nanocrystalline domains, the charge transfer efficiency was improved, and the stability of the hydrogel interface was enhanced concurrently. The sensitivity of this sensor to PCN in complex biological matrices, including PBS, LB broth, and saliva, is enhanced by 73% when compared to that in a single-material system. In the supernatant of LB broth where PA has been cultured, the deviation between the PCN detection results obtained by this sensor and those of the standard method is less than 0.2%, thereby validating its reliability in complex biological settings. Furthermore, this sensor achieves the functional extension of multi-component detection for non-specific sensors. The response stability (RSD < 6%) and rapid detection capacity (within 4 min) in the presence of coexisting substances satisfy the requirements of clinical point-of-care testing, laying a technical foundation for the development of wound infection POCT devices with clinical practical significance.

## Data Availability

All data generated or analyzed during this study are included in this article or Supplementary Material.

## Supplementary Materials

The following supporting information can be downloaded at: https://www.mdpi.com/article/10.3390/mi17020209/s1, Figure S1: Preparation of MWCNTs/MXene/PVA series sensors and their structural schematic diagrams; Figure S2: A series of MWCNTs dispersions (A) Schematic diagram and photo of the preparation; (B) UV-Vis absorption spectrum. (C) The normalized absorbance at 262 nm (A/A_0_) as a function of time under different CTAB concentrations; Figure S3: (A) CV curves of MWCNTs0.6/MXene0.4/PVA in a 50 μM PCN environment at various scan rates; (B) The correlation between the scan rate and the anodic and cathodic peak currents of the CV curves; Figure S4: MWCNTs0.6/MXene0.4/PVA sensor(A) Anti-interference capacity; (B) Stability; (C) Reproducibility; (D) Long-term operational stability; Figure S5: The sensor prepared from the upper solution of MWCNTs0.6/MXene0.4/PVA (A) SEM image at a scale of 5 μm, (B) SEM image at a scale of 1 μm, and (C) EDS surface scanning image; Figure S6: SEM images of MWCNTs/MXene/PVA series sensors (A) MWCNTs0.9/MXene0.1/PVA (B) MWCNTs0.6/MXene0.4/PVA (C) MWCNTs0.5/MXene0.5/PVA; Figure S7: XPS survey spectra of the MWCNTs0.6/MXene0.4/PVA and MWCNTs0.9/MXene0.1/PVA sensors; Figure S8: The Randle equivalent circuit diagram of the EIS of MWCNTs/MXene/PVA series sensors and the fitting parameters of each element in the equivalent circuit; Figure S9. MWCNTs0.6/MXene0.4/PVA (A) CV curves at different scan rates in the environment of 500 μM UA; (B) The relationship between the scan rate and the anodic peak current of CV; (C,D) SWV current response in PBS solution and its standard curve; Figure S10: MWCNTs0.6/MXene0.4/PVA (A) CV curves at different scan rates in a 50 μM HA environment; (B) The relationship between the scan rate and the anodic and cathodic peak currents in the CV; (C,D) The SWV current response in PBS solution and its standard curve; Figure S11: MWCNTs0.6/MXene0.4/PVA (A) SWV current responses under different conditions; (B) SWV current responses with a fixed UA concentration and varying HA concentrations; (C,D) SWV current responses with a fixed HA concentration and gradient changes in UA concentration, and their standard curves; Figure S12: MWCNTs0.6/MXene0.4/PVA (A) SWV current responses under different conditions; (B) SWV current responses with a fixed UA concentration and varying PCN concentrations; (C,D) SWV current responses with a fixed PCN concentration and gradient changes in UA concentration, and their standard curves; Figure S13: Current response of the MWCNTs/MXene/PVA sensor series to 50 μM PCN under different freeze-thaw cycles; Table S1: Percentage content of various types of C in MWCNTs/MXene/PVA series sensors; Table S2: Comparison of the performances of the MWCNTs0.6/MXene0.4/PVA sensor with those of other sensors.
