# Supplementary material for: Development of MWCNTs/MXene/PVA Hydrogel Electrochemical Sensor for Multiplex Detection of Wound Infection Biomarkers
Source: Micromachines (Basel). 2026 Feb 3;17(2):209. doi: 10.3390/mi17020209 (PMC12943359; doi:10.3390/mi17020209)
Supplement: Supplementary file 1 [file micromachines-17-00209-s001.zip › micromachines-4104528-supplementary.pdf]

SUPPLEMENTARY METHODS

Materials

Carboxylated multi-walled carbon nanotubes (>50 nm) were purchased from Jiangsu XianFeng Nano Technology Co.,Ltd.,China.; MXene were purchased from Xi'an Qiyue Biological Technology Co.,Ltd.; Hexadecyl trimethyl ammonium Bromide(CTAB), poly(vinyl alchol)(PVA, MW 146000-186000,with 99% degree of hydrolysis), pyocyanin CRS, were obtained from Aladdin Chemical Reagent Co., Ltd., China. Pyocyanin sample purified from P. aeruginosa. All solutions were prepared using Milli-Q grade water (Millipore water purification system Z18 MΩ, Milli-Q, Millipore, Billerica, MA).

SUPPLEMENTARY FIGURES

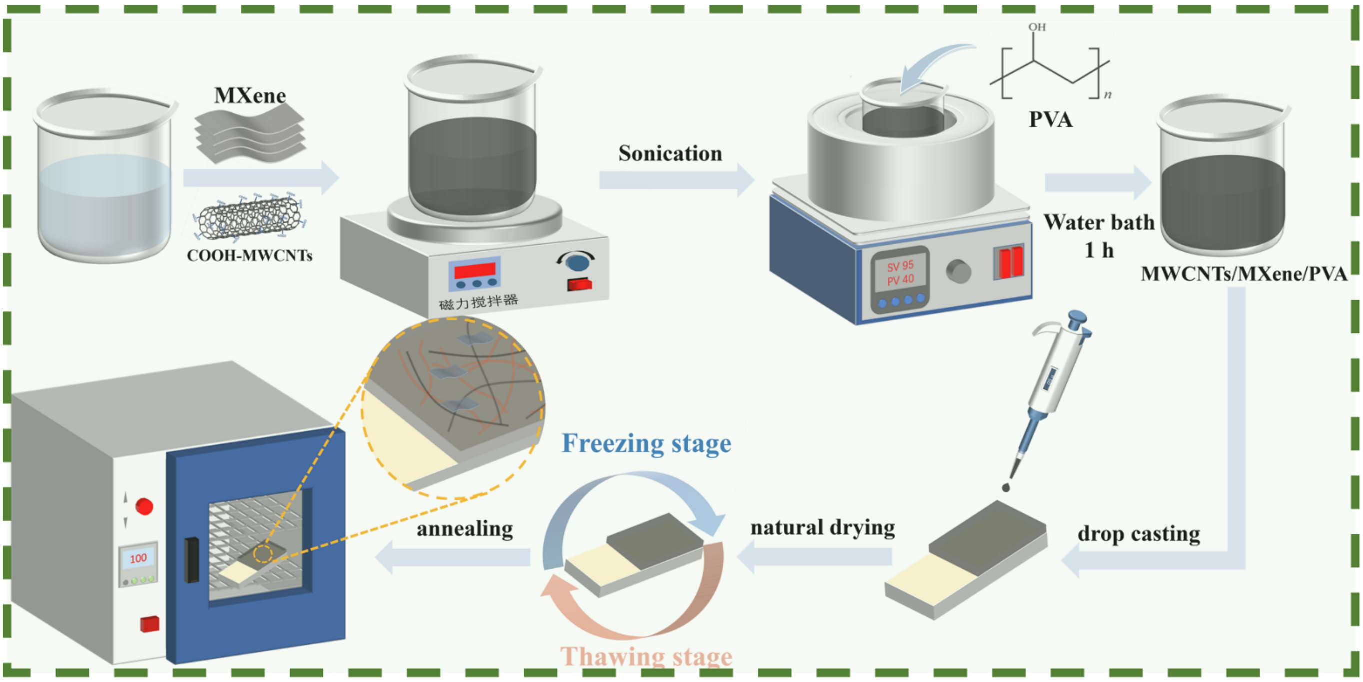

Figure S1. Preparation of MWCNTs/MXene/PVA series sensors and their structural schematic diagrams.

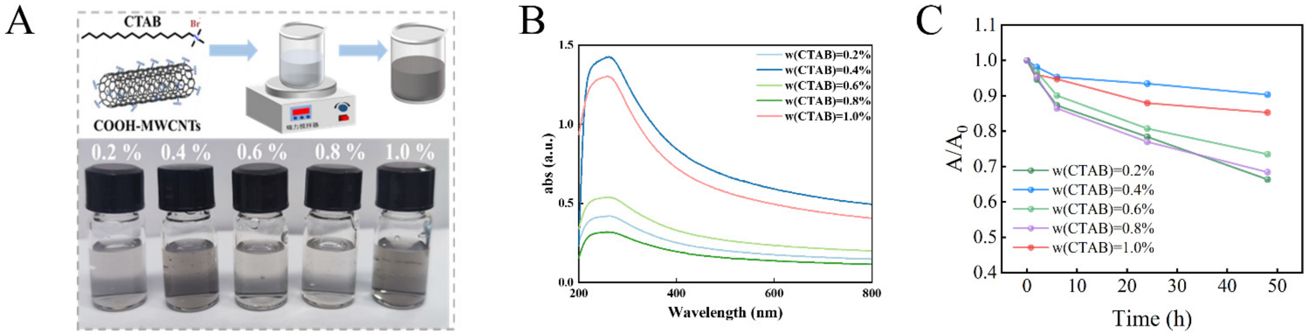

Figure S2. A series of MWCNTs dispersions (A) Schematic diagram and photo of the preparation; (B) UV-Vis absorption spectrum. (C) The normalized absorbance at 262 nm (A/A_0) as a function of time under different CTAB concentrations

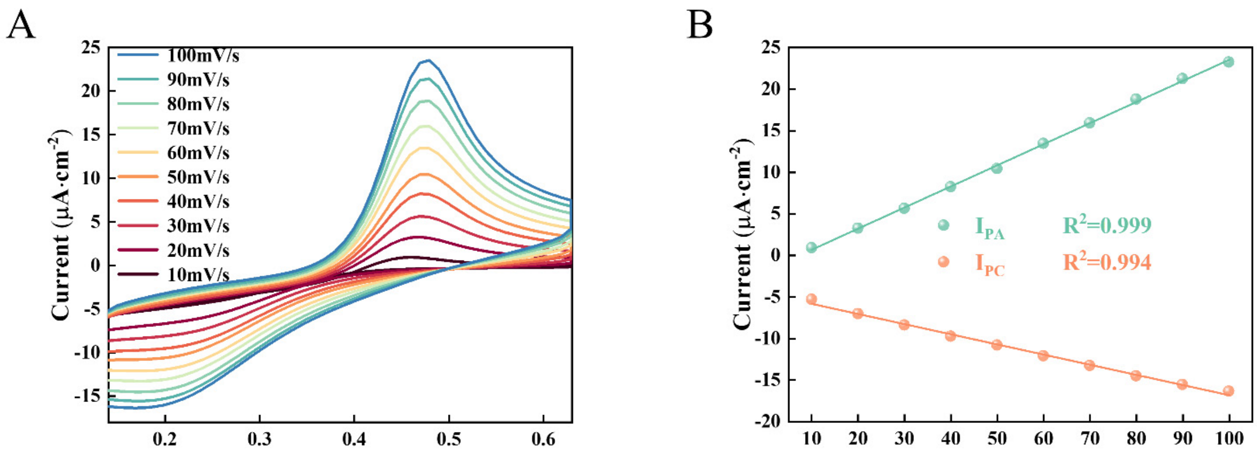

Figure S3. (A) CV curves of $MWCNTs_{0.6}/MXene_{0.4}/PVA$ in a 50 μM PCN environment at various scan rates; (B) The correlation between the scan rate and the anodic and cathodic peak currents of the CV curves.

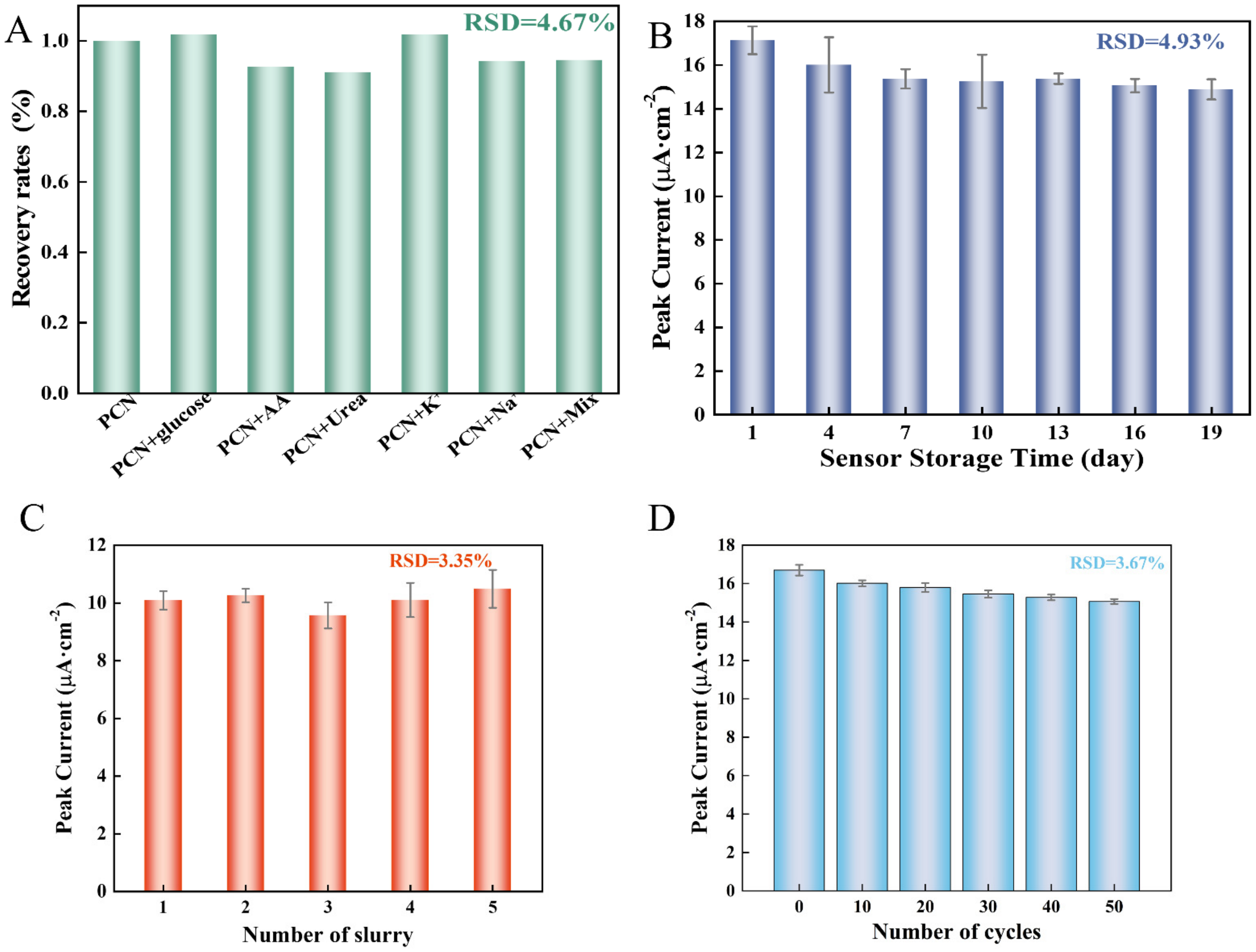

Figure S4. $MWCNTs_{0.6}/MXene_{0.4}/PVA$ sensor(A) Anti-interference capacity; (B) Stability; (C) Reproducibility; (D) Long-term operational stability .

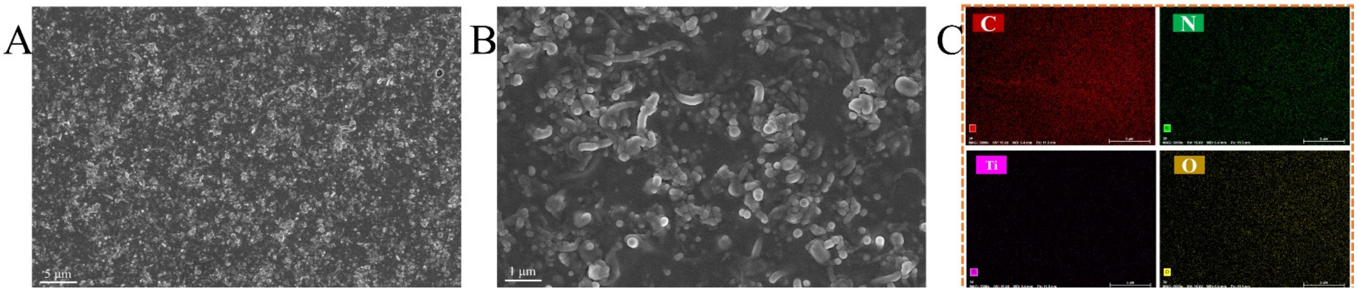

Figure S5. The sensor prepared from the upper solution of $MWCNTs_{0.6}/MXene_{0.4}/PVA$ (A) SEM image at a scale of 5 μm, (B) SEM image at a scale of 1 μm, and (C) EDS surface scanning image.

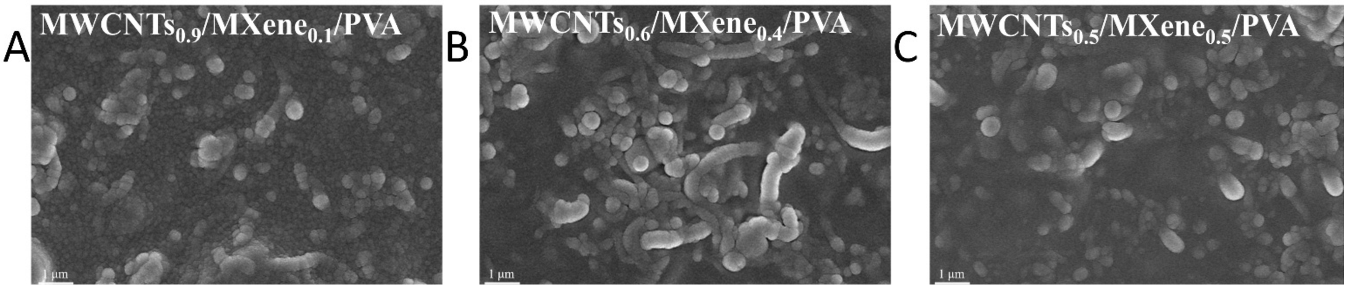

Figure S6. SEM images of MWCNTs/MXene/PVA series sensors (A) $MWCNTs_{0.9}/MXene_{0.1}/PVA$ (B) $MWCNTs_{0.6}/MXene_{0.4}/PVA$ (C) $MWCNTs_{0.5}/MXene_{0.5}/PVA$.

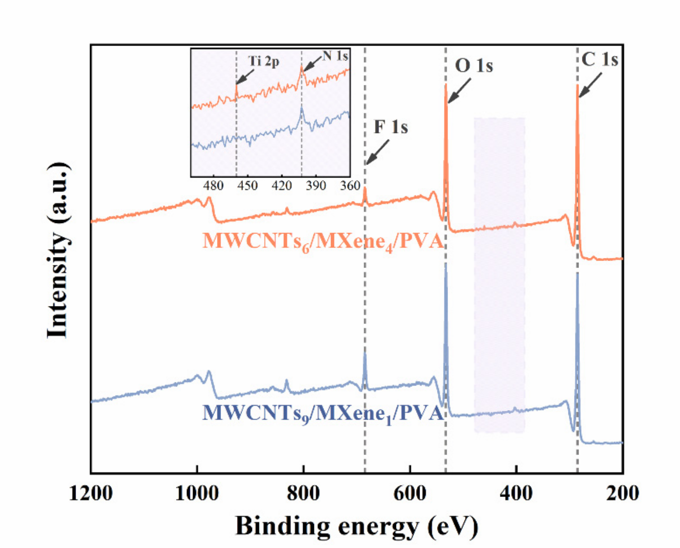

Figure S7. XPS survey spectra of the $MWCNTs_{0.6}/MXene_{0.4}/PVA$ and $MWCNTs_{0.9}/MXene_{0.1}/PVA$ sensors.

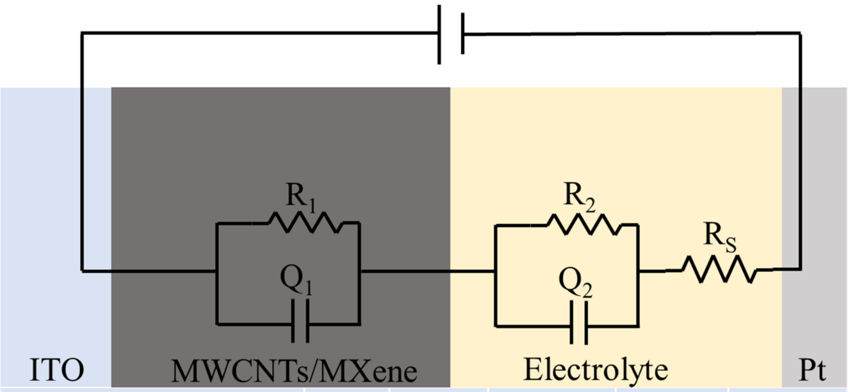

Sensor system	R_S (Ω)	R_1 (Ω)	$Q_1(S^{-SEC})$	R_2 (Ω)	$Q_2(S^{-SEC})$
MWCNTs/PVA	102.8	122.4	0.000061	133.9	0.000005
$MWCNTs_{0.9}/MXene_{0.1}/PVA$	100.6	112.6	0.000016	140.3	0.000046
$MWCNTs_{0.6}/MXene_{0.4}/PVA$	99.44	87.79	0.000010	101.6	0.000051
$MWCNTs_{0.5}/MXene_{0.5}/PVA$	104.5	96.27	0.000008	136.3	0.000037

Figure S8. The Randle equivalent circuit diagram of the EIS of MWCNTs/MXene/PVA series sensors and the fitting parameters of each element in the equivalent circuit.

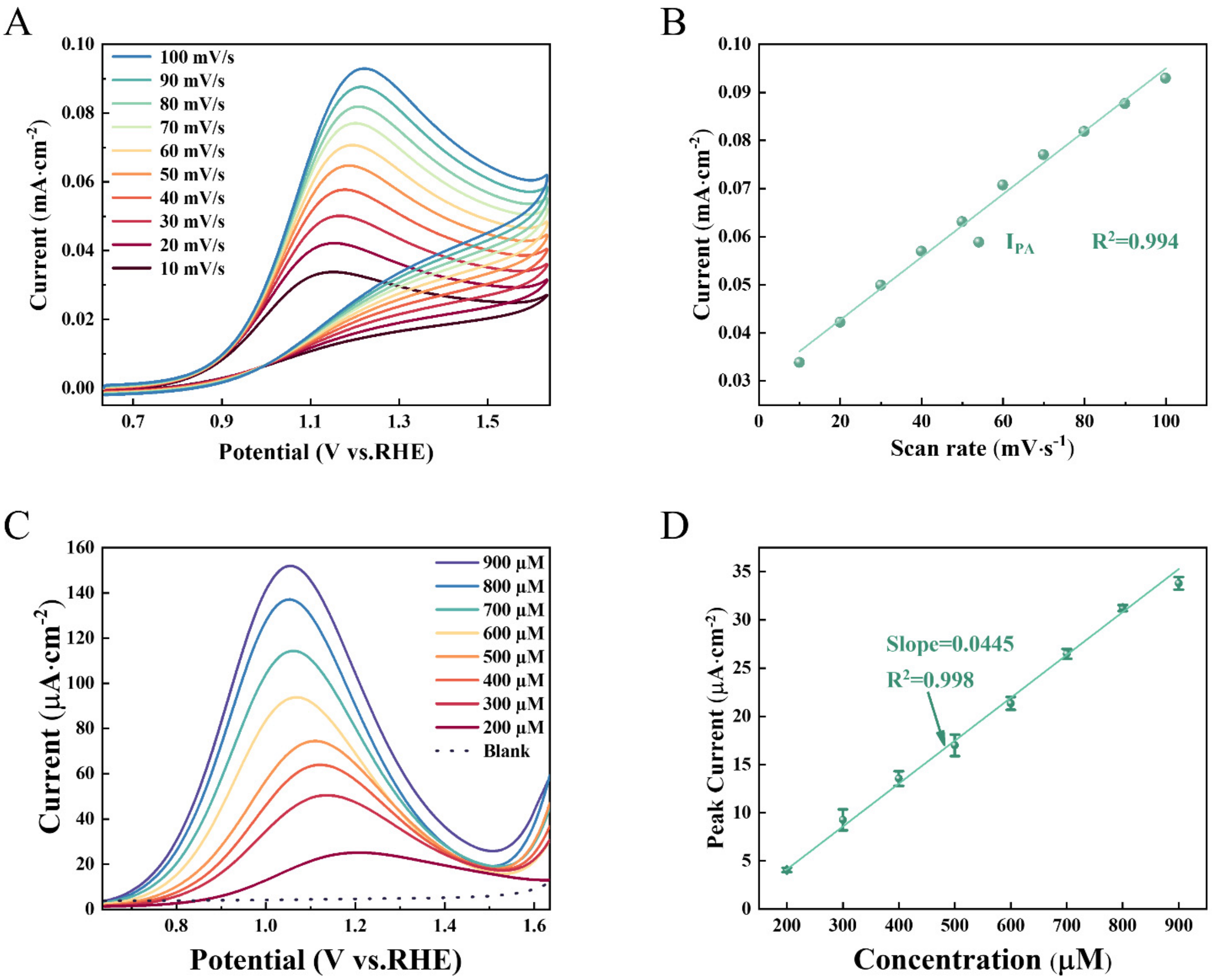

Figure S9. $MWCNTs_{0.6}/MXene_{0.4}/PVA$ (A) CV curves at different scan rates in the environment of 500 μM UA; (B) The relationship between the scan rate and the anodic peak current of CV; (C-D) SWV current response in PBS solution and its standard curve.

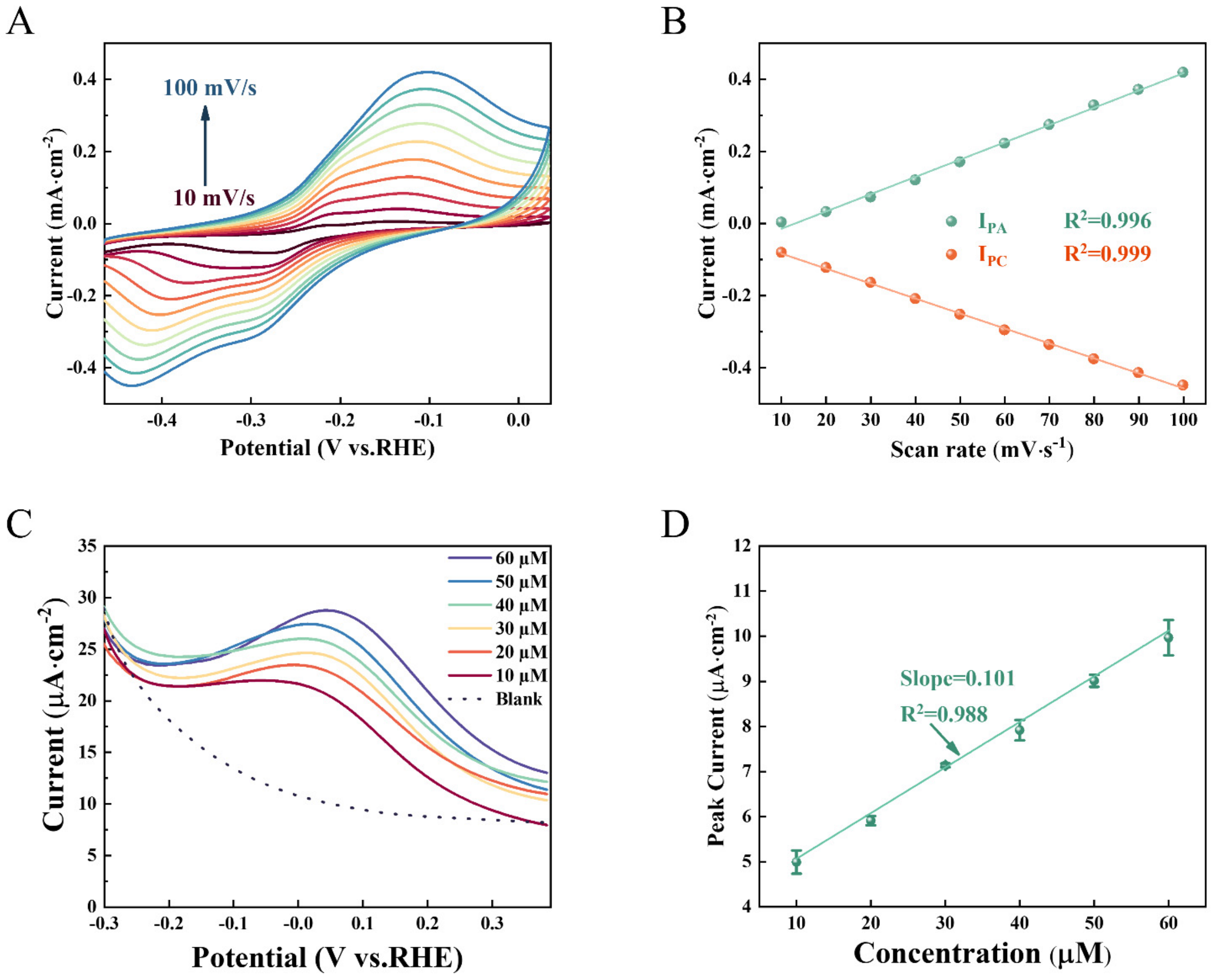

Figure S10. $MWCNTs_{0.6}/MXene_{0.4}/PVA$ (A) CV curves at different scan rates in a 50 μM HA environment; (B) The relationship between the scan rate and the anodic and cathodic peak currents in the CV; (C-D) The SWV current response in PBS solution and its standard curve.

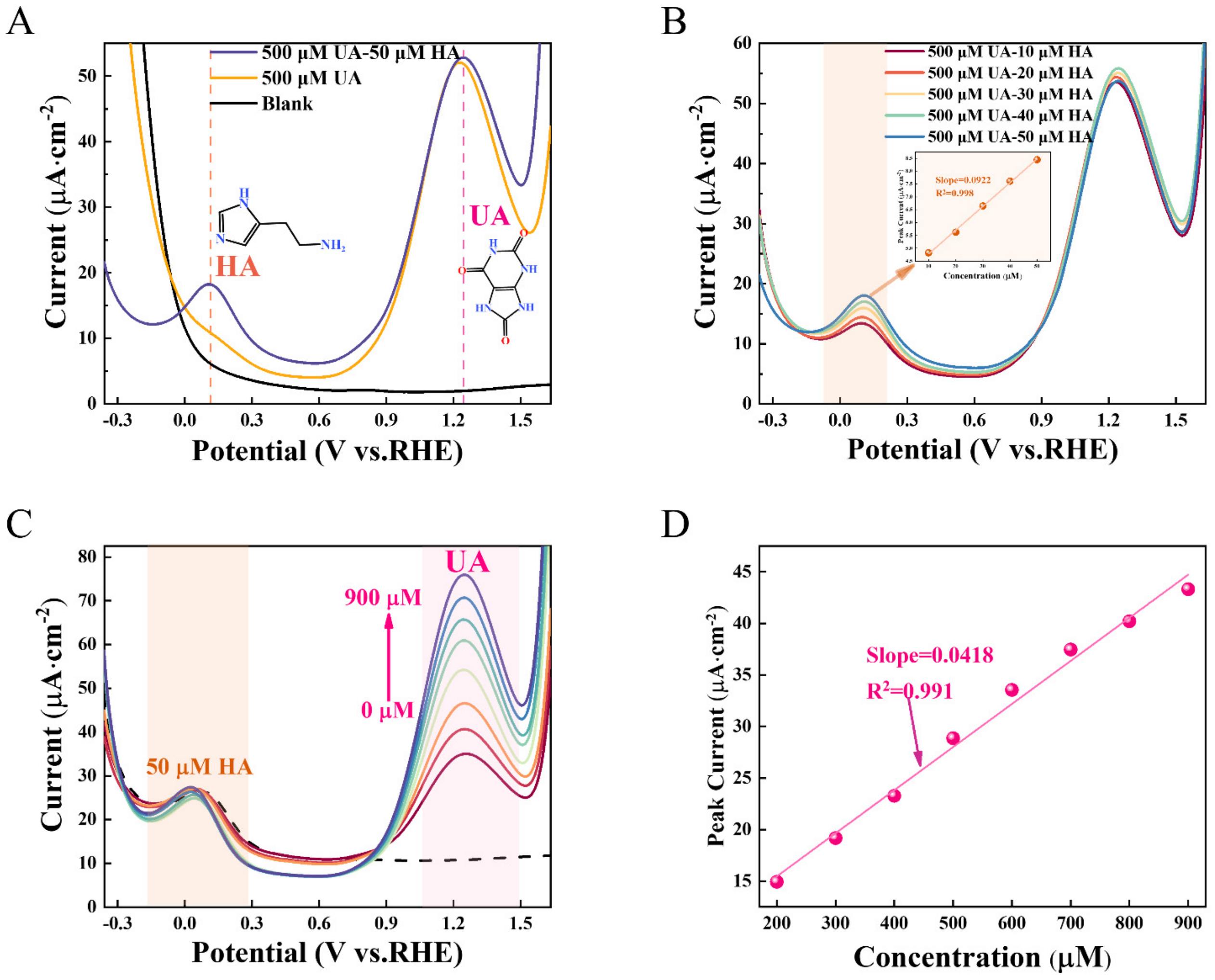

Figure S11. MWCNTs$_{0.6}$/MXene$_{0.4}$/PVA (A) SWV current responses under different conditions; (B) SWV current responses with a fixed UA concentration and varying HA concentrations; (C-D) SWV current responses with a fixed HA concentration and gradient changes in UA concentration, and their standard curves.

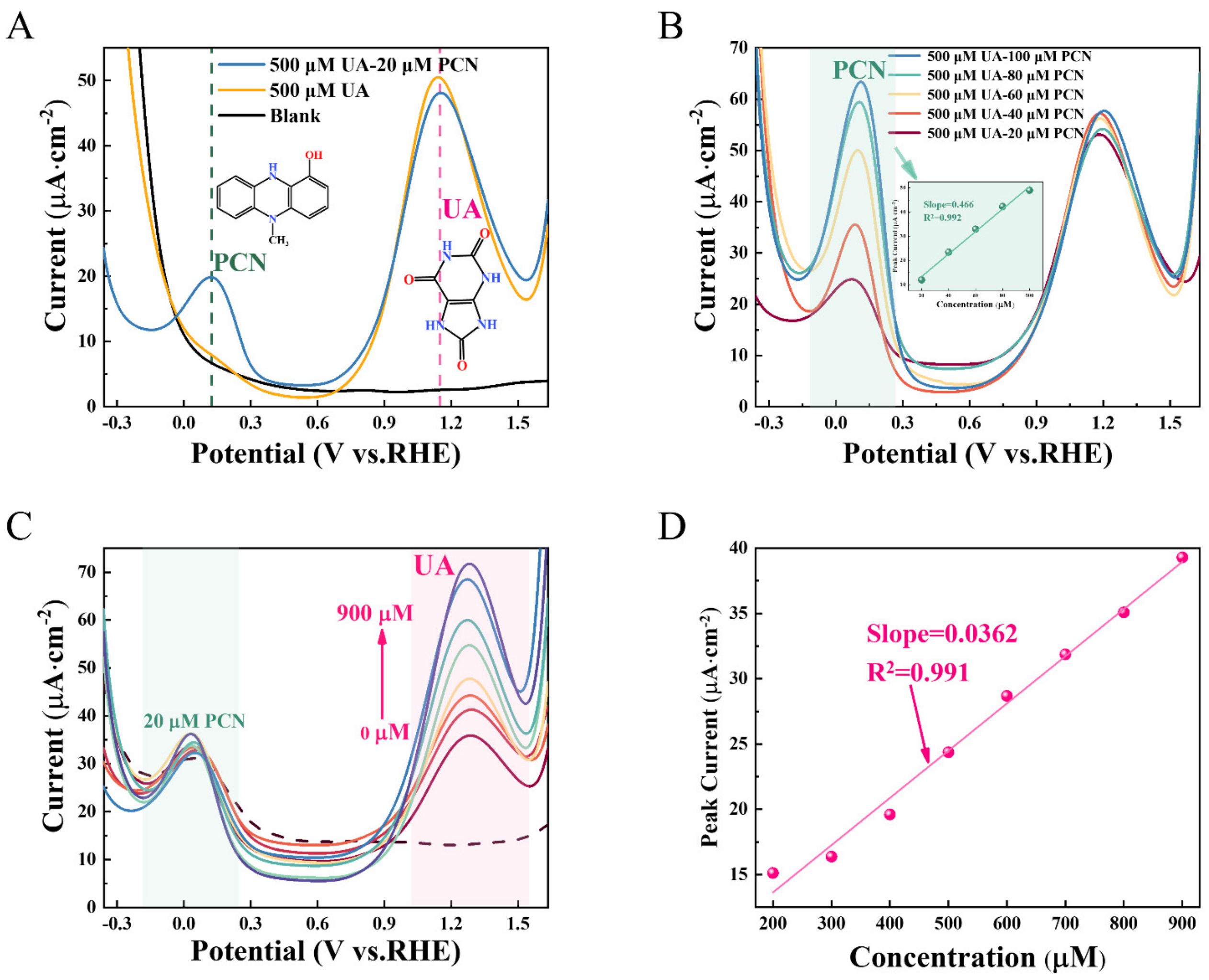

Figure S12. MWCNTs$_{0.6}$/MXene$_{0.4}$/PVA (A) SWV current responses under different conditions; (B) SWV current responses with a fixed UA concentration and varying PCN concentrations; (C-D) SWV current responses with a fixed PCN concentration and gradient changes in UA concentration, and their standard curves.

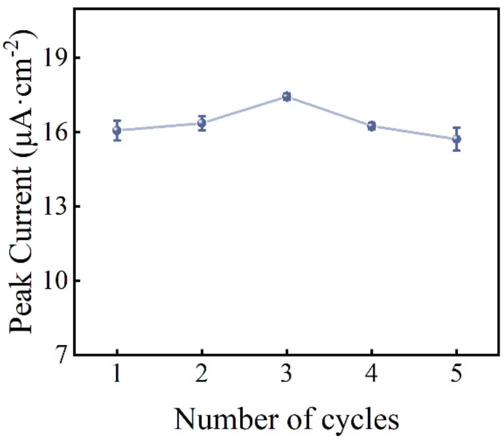

Figure S13. Current response of the MWCNTs/MXene/PVA sensor series to 50 μM PCN under different freeze-thaw cycles.

SUPPLEMENTARY TABLE

Sensing system	C-C(%)	C-O(%)	C=O(%)	C-Ti-O(%)
$MWCNTs_{0.9}/MXene_{0.1}/PVA$	49.62	42.38	4.55	3.45
$MWCNTs_{0.6}/MXene_{0.4}/PVA$	46.85	41.66	4.49	7.01

Table S1. Percentage content of various types of C in MWCNTs/MXene/PVA series sensors.

Sensor	Analytes	LOD/ Linear Range (μm)	Detection time	POCT	Advantage	Limitations
MIPs/AuNPs/SPCE[11]	PCN	0.74μm /1-100μm	20min	High selectivity	Low cost and high selectivity	Only a single analyte
$MWCNTs_{0.6}/MXene_{0.4}/PVA$	PCN<br>UA<br>HA	PCN:0.94μm/5-100μm<br>UA:66.7μm/200-900μm<br>HA:3.34μm/10-60μm	5min	Fast, low-cost, multiple analytes, minimum sample pretreatment	Low cost, multiple analytes, less sample pretreatment	The detection range is slightly narrower
Ti3C2Tx-PEGDA hydrogel/Au[23]	DA<br>UA	DA: 2.55 μM/ 2.5–200 μM<br>UA: 25.11 μM/10–100 μM	/	Limited detection range	Low cost, multiple analytes	The detection range of the UA test did not cover the clinically relevant concentrations

Table S2. Comparison of the performances of the $MWCNTs_{0.6}/MXene_{0.4}/PVA$ sensor with those of other sensors.
